# Situational perception in distracted driving: an agentic multi-modal LLM framework

**DOI:** 10.3389/frai.2025.1669937

**Published:** 2025-10-15

**Authors:** Ahmad M. Nazar, Mohamed Y. Selim, Ashraf Gaffar, Daji Qiao

**Affiliations:** Department of Electrical and Computer Engineering, Iowa State University, Ames, IA, United States

**Keywords:** LLM, distracted driving, multi-modal, LLM agents, data-driven, situational awareness, perception

## Abstract

**Introduction:**

Distracted driving is a significant public safety concern, causing thousands of accidents annually. While most driver assistance systems emphasize distraction detection, they fail to deliver real-time environmental perception and context-aware interventions.

**Methods:**

We propose a large language model (LLM)-driven intervention framework that assumes distraction is pre-detected and dynamically integrates camera and GPS inputs to generate verbal driver alerts. The framework employs an agentic design, where specialized tools handle object detection, speed limits, live traffic conditions, and weather data. Structured orchestration ensures information is fused efficiently, balancing accuracy with conciseness to avoid overwhelming the driver.

**Results:**

Evaluation of the system demonstrates high performance, with semantic intervention correctness of 85.7% and an average response latency of 1.74 s. Compared to conventional ML-based driver assistance approaches, our framework effectively synthesizes multi-modal environmental data and produces actionable alerts in real time.

**Discussion/conclusion:**

These findings highlight the potential of LLM-driven, multi-modal reasoning for distracted driving intervention. Integrating specialized agents and structured orchestration improves situational awareness, maintains concise communication, and meets real-time safety requirements. This proof-of-concept establishes a pathway for deploying intelligent, AI-driven driver support systems in safety-critical applications.

## 1 Introduction

Distracted driving is a critical public safety issue, causing approximately eight deaths and over 1,000 injuries daily in the United States (The Law Office of Melinda J. Helbock A.P.C., 2024). Despite advancements in driver technologies, distraction-related crashes persist due to the increased use of mobile devices, and other cognitive distractions. While conventional monitoring systems can detect distraction, they often lack proactive intervention mechanisms (National Center for Statistics and Analysis, 2020; World Health Organization, 2015).

Traditional machine learning (ML)-based driver assistance systems mainly detect visual distractions through in-cabin monitoring using vision-based models. However, they often fail to account for external environmental factors, limiting their ability to generate context-aware interventions. Conventional ML models rely on static datasets and predefined heuristics, making it challenging to integrate real-time multi-modal data such as object detection and road conditions (Cui et al., 2024).

In contrast, large language model [large language models (LLMs)] provide a more adaptive approach to real-time decision-making by integrating an agentic reasoning framework with structured prompt engineering. They dynamically query external APIs and synthesize sensor inputs to generate contextually relevant responses (Jin et al., 2024; Gu et al., 2023). These models have shown potential as decision-making evaluators while effectively processing multi-modal data for situational awareness (Gu et al., 2025).

LLM-driven frameworks are also effective in autonomous driving, where multi-modal architectures improve situational awareness and response time. LLMs' ability to process multi-modal data ensures a more comprehensive situational awareness. For instance, Waymo's model fuses multi-modal data for real-time decision-making, outperforming traditional systems in complex tasks and mimicking human-like driving behavior with contextual reasoning (Hwang et al., 2024; Kim and Park, 2025; Fu et al., 2024; Zhou et al., 2024).

Beyond perception, LLMs enable context-aware driving actions through natural language commands, enhancing environmental awareness and driver reaction time (Nguyen et al., 2024; Zhu et al., 2025). Structured LLM orchestration using agentic frameworks ensures reliable, context-grounded reasoning for real-time decision-making in intelligent driver assistance systems (Talebirad and Nadiri, 2023; Jin et al., 2024).

This work introduces an LLM-driven intervention framework for distracted driving, emphasizing real-time environmental perception and driver re-engagement after distraction detection. The system dynamically activates an LLM to invoke specialized agents for object detection, retrieve speed limits, traffic conditions, and weather awareness, and generate interventions according to structured tasks.

The primary contributions of this work include the development of an LLM-driven intervention framework that dynamically integrates awareness agents to deliver real-time driver alerts post-distraction detection. The framework features a multi-modal environmental perception pipeline that combines YOLO-based vision analysis, speed limit retrieval, live traffic monitoring, and weather assessment. The system employs a structured LLM orchestration framework supported by LlamaIndex. The framework achieved 85.3% semantic correctness and a 1.74s response time, meeting the sub-2s latency requirement for practical driver assistance.

The paper is organized as follows: Section 2.1 provides an overview of the system architecture. Section 2.2 describes the LLM orchestration methodology. Section 3 presents the evaluation and results, followed by case studies in Section 3.7. Finally, Section 4 discusses conclusions and future work.

## 2 Materials and methods

### 2.1 Framework overview

**Figure 1:**
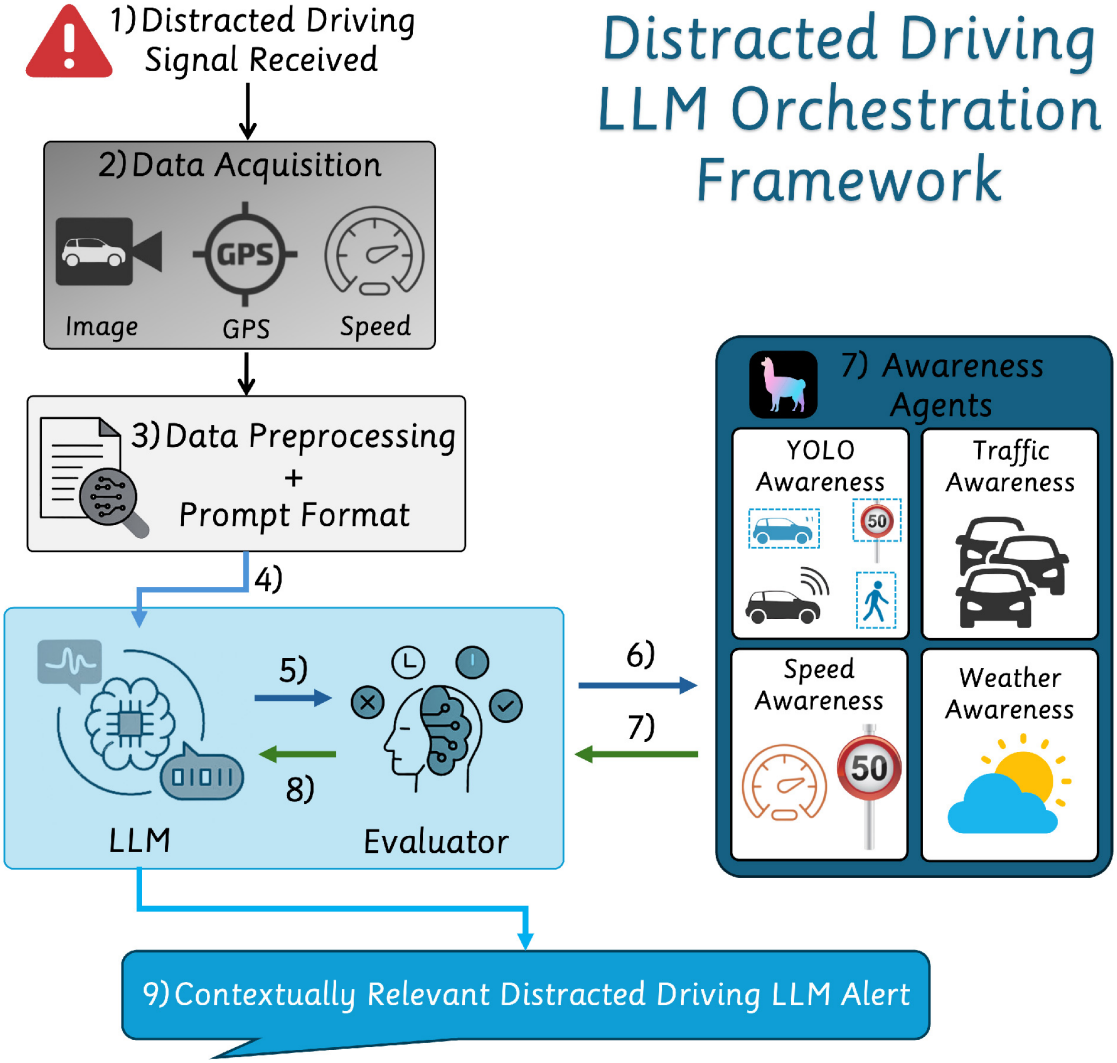
Overview of the proposed framework with the LLM's agent orchestration process, data acquisition pipeline, multi-modal intervention generation, and an agentic evaluator and invoker.

The proposed framework, as shown in Figure 1, is activated upon detecting a distracted driver trigger signal, gathering real-time multi-modal data from a GPS module and a camera. It employs an LLM-driven decision pipeline to integrate perception, speed limit awareness, weather analysis, and traffic congestion data. At its core, the system utilizes an LLM-orchestrated framework to invoke awareness agents based on the contextual driving scenario dynamically. Upon receiving sensor data, the LLM evaluates the environmental context and identifies necessary data inputs, utilizing YOLO-based object detection Khanam and Hussain (2024) and external APIs such as HERE^1^, OpenMeteo^2^, and OpenStreetMap Overpass^3^. The system dynamically structures the extracted data to generate context-aware driver interventions.

Upon detecting driver inattention, the system collects GPS coordinates, speed, and driving scene images. After preprocessing and structuring this data into a task-oriented prompt, the LLM initiates decision-making. The processed inputs are sent to the agent evaluator, which selects appropriate agents based on data completeness and context. This process iterates as needed to gather further contextual information.

Once all relevant data is gathered, the LLM synthesizes the information into a concise, contextually appropriate interventions alert. The final intervention is delivered as **clear auditory feedback** to help the driver regain focus.

#### 2.1.1 Datasets

To ensure robust detection of vehicles, pedestrians, road signs, and environmental hazards, we utilize two datasets: DeepSense6G (Scenarios 36–39) (Charan et al., 2022; Alkhateeb et al., 2023), and the LISA traffic sign dataset (Mogelmose et al., 2012).

The DeepSense6G dataset is a multi-modal dataset captured from an outdoor real-world vehicle-to-vehicle (V2V) communications environment, and includes time-synchronized camera, GPS, LiDAR, and radar data from various driving scenarios with diverse characteristics. We explicitly frame our study as a proof-of-concept, while our use of real-world multimodal V2V datasets provides a realistic proxy for deployment conditions. Our system uses camera images and GPS data for road condition analysis. The LISA traffic dataset is used to train a fine-tuned YOLOv11 model specializing in recognizing road symbols common to USA roadways.

#### 2.1.2 Data acquisition

After detecting a distracted driver signal, the system automatically gathers data from GPS and camera sensors. The GPS module records real-time location coordinates, while the onboard camera captures high-resolution roadway images, which are resized to (640 × 640). The GPS data is preprocessed to extract latitude, longitude, and speed. Although real-world deployment would involve continuous sensor input from an active vehicle, our evaluation uses extracted data from the aforementioned datasets to ensure consistency during testing.

#### 2.1.3 Grounding and prompt engineering

The system employs structured agent invocation and contextual tracking to ensure factual accuracy and contextual relevance, guided by carefully designed prompts. The LLM is specifically configured to utilize only data from designated agents, thereby minimizing speculative or hallucinated responses.

Preprocessed inputs are formatted within an instructional prompt template that clearly defines the LLM's role as an assistant invoked only upon detecting distracted driving events. This prompt explicitly mandates using available awareness agents to extract data from the environment and synthesize responses based solely on verified outputs. For instance, the template enforces that the model must not fabricate content if an agent (e.g., traffic or speed limit awareness agent) does not return information. This instructional template also enforces the maximum number of times (once) an agent is invoked to eliminate redundant or recursive agent calls. Additionally, it requires the LLM to provide a structured explanation of its reasoning via bullet points, enforcing transparency and traceability of decisions.

This structured prompt ensures the LLM follows a strict reasoning sequence: assess object detections, selectively invoke agents (at most once) as needed, and synthesize a concise, alert-style summary grounded entirely in retrieved sensor or API outputs. By defining functional expectations and behavioral constraints, the grounding strategy constrains the generative space of the model, significantly reducing hallucination and ensuring the intervention remains reliable, explainable, and situationally appropriate.

#### 2.1.4 Awareness agents

The framework integrates multiple awareness agents specializing in environmental perception to maintain situational accuracy. These agents work collaboratively to process multi-modal data, minimizing redundant computations. The system dynamically invokes the required agents based on detected driving conditions. The awareness agents cover two significant aspects: Road Perception and Weather Awareness.

##### 2.1.4.1 Road perception awareness

This multi-modal system fuses YOLO-based object detection with geospatial data to identify key road elements, including vehicles, pedestrians, regulatory signs, and hazards. Speed limit awareness is achieved through dual-mode detection: using YOLO for physical signs and querying OpenStreetMap when signage is not visible. The system cross-references speed data with the vehicle's GPS-derived speed, adjusting intervention severity based on road and weather conditions.

##### 2.1.4.2 Weather awareness

Using the Open-Meteo API, the system retrieves real-time weather data to assess driving risks, such as reduced visibility, slippery roads, or high winds.

#### 2.1.5 LLM awareness orchestration framework

The LLM-driven agent orchestration dynamically selects and executes relevant awareness agents based on the detected distraction. The LLM assesses which data is missing and instructs the agent evaluator to call the relevant agent. The evaluator prioritizes agents based on their relevance to the current context, ensuring accurate data retrieval.

The LLM synthesizes a structured intervention upon gathering the necessary data, ensuring that responses remain grounded and factually accurate. This dynamic process allows the system to adapt to changing road conditions, providing precise and situationally relevant interventions. Once the system compiles the intervention, it delivers the response via a text-to-speech mechanism to verbally alert the driver, ensuring immediate and clear communication.

### 2.2 LLM awareness orchestration framework

A central innovation of this system is utilizing the LLM as the core reasoning engine, synthesizing multi-modal inputs from object detection, traffic sign recognition, weather conditions, and real-time traffic data. LlamaIndex is an integration layer, setting up the structured space to interact with dedicated agents. However, the primary evaluation, assessment, and decision-making are driven by the LLM itself. The LLM dynamically identifies missing information in the current environmental context and instructs the agent evaluator to invoke the appropriate agent, leveraging task descriptions provided as metadata to both the LLM and the evaluator.

#### 2.2.1 LLM-driven decision framework

The system follows a structured instruction template to ensure a systematic decision-making process. The LLM initially assesses the available environmental data and identifies any missing information needed to make a contextually relevant decision. The agent evaluator then takes the required inputs and calls the corresponding agent to retrieve the missing data.

The process begins when the LLM receives preprocessed GPS and image data. The LLM first evaluates the completeness of the contexts by checking for missing or ambiguous information. For instance, if the LLM detects that a speed limit is unavailable from visual inputs (e.g., YOLO not sensing a speed limit), it flags the absence of speed data.

Once a gap is identified, the LLM determines which specific data is required and instructs the agent evaluator to invoke the appropriate agent. The evaluator then assesses the available agents and selects the most likely to fill the data gap. For example, in the case of missing speed limit data, the evaluator calls the speed limit awareness agent, which queries OpenStreetMap to retrieve local speed limits.

To ensure grounding and mitigate against redundant or unnecessary agent calls, our framework enforces an instruction-grounded template that conditions the LLM to invoke agents selectively. Agent calls are not arbitrarily triggered; rather, they are embedded within a structured reasoning workflow that encourages the LLM to synthesize available perceptual cues (e.g., from YOLO) before invoking external sources. While agent queries may still occur even if partial data is present, this is intentional: agents are used as supplementary sources when available to enrich the system's situational awareness, not merely as last-resort fallbacks. For example, OpenStreetMap may be queried to cross-check speed limits even if a sign is detected, enhancing robustness against visual occlusions or detection failures. This optional-but-informed invocation policy helps balance reliability and completeness.

The agent evaluator follows a priority-based decision tree where agents are ranked according to their ability to provide reliable data for a given context. If the initial agent does not return adequate information or the data remains incomplete, the evaluator may attempt secondary agents or cross-reference outputs. This validation ensures robustness and contextual accuracy in the generated response. Once collected, the LLM synthesizes these contexts, integrating object detections, traffic signs, weather conditions, and speed regulations to determine the optimal intervention strategy. This dynamic agent selection process ensures that the system efficiently retrieves all relevant and necessary contexts.

Unlike conventional decision-making models, the LLM operates within a constrained reasoning framework where each decision is grounded in real-time environmental inputs. This approach ensures that every response is data-driven, reducing reliance on assumptions or speculative reasoning. The structured multi-step process prevents hallucinations by requiring the LLM to rely exclusively on retrieved agent outputs, ensuring that all interventions are factually supported.

#### 2.2.2 Agent implementation

Each agent is designed and implemented according to the relevant functionality, and their invocation logic is directly handled within the framework. The LLM evaluates the context and determines the needed data. If data is missing or incomplete, the LLM instructs the agent evaluator to invoke the appropriate agent to retrieve relevant data. The framework includes four primary agents with distinct tasks:

1. *YOLO Awareness Agent* processes onboard camera images to detect road elements, including obstacles, pedestrians, vehicles, speed limits, stop signs, and other regulatory signage. The model is fine-tuned using augmented training data, with augmentations such as random rotations, horizontal flips, brightness/contrast adjustments, and added Gaussian noise, ensuring robustness across varying perceived conditions. After detection, this agent estimates object proximity and density by analyzing dynamic object classes, such as vehicles and cyclists, by measuring pixel-wise distances and bounding box overlaps. If the number of detected dynamic objects exceeds a threshold and spatial proximity falls below a predefined distance margin, the system flags the environment congested, enabling the evaluator to infer traffic presence and busy road conditions.
2. *Speed Limit Awareness Agent* queries OpenStreetMap to retrieve GPS-based legal speed limits, complementing YOLO's traffic sign recognition by providing speed regulations when physical signs are not detected. This agent confirms whether the vehicle is currently over the speed limit or safely within its limits
3. *Traffic Awareness Agent* leverages the HERE Traffic API to assess real-time congestion levels, identifying potential slowdowns due to traffic conditions.
4. *Weather Awareness Agent* retrieves meteorological data from Open-Meteo, incorporating weather conditions into the decision-making process to ensure safe driving recommendations under varying environmental conditions.

Each agent in the system operates independently yet in coordination, contributing to a dynamic and context-aware response mechanism. The primary mode of perception and reasoning is driven by the onboard YOLO-based vision awareness agent, which handles core tasks such as obstacle detection, traffic sign recognition, and environmental layout understanding. External APIs are invoked to supplement the YOLO awareness agent and serve as complementary data sources rather than critical dependencies. For example, suppose a speed limit sign is not detected visually due to occlusion or noise. In that case, the LLM prompts the agent evaluator to query OpenStreetMap to supplement the missing contextual information. Similarly, traffic congestion detected via dense bounding box overlap by YOLO can be further contextualized by querying HERE to determine the underlying cause (e.g., construction, accidents).

This tiered design ensures robustness: even if API endpoints are temporarily inaccessible, the system can still generate meaningful interventions based on YOLO detections and past context. Thus, the framework balances autonomy and augmentation to ensure real-time operation with graceful degradation in resource-constrained or disconnected environments.

#### 2.2.3 Contextual reasoning and grounded decision making

A critical aspect of the LLM's operation is ensuring that all generated interventions remain grounded in real-time captured data rather than speculative assumptions. The LLM achieves this by dynamically incorporating agent outputs into its decision-making process. The structured instruction template explicitly directs the LLM to invoke necessary agents first, extract relevant data, and only then synthesize an intervention.

Using agent outputs as contextual anchors, the LLM ensures that every response accurately reflects the driving environment. For example, if the weather API detects fog and the YOLO model identifies a pedestrian near a crosswalk, the LLM explicitly references both risk factors in its generated intervention. This structured methodology prevents ambiguous recommendations and reinforces fact-based decision-making.

Strict adherence to function signatures within LlamaIndex also ensures that agent calls remain valid and correctly formatted. The LLM is constrained to operating only within its agents, eliminating the risk of extraneous or hallucinated responses. In cases where agent outputs are unavailable, the system gracefully handles missing data by adjusting its reasoning process or prioritizing other environmental factors.

#### 2.2.4 Dynamic reasoning and alert generation

Once the necessary data is extracted, the LLM synthesizes a structured intervention consisting of three components: an environmental summary, a risk factor analysis, and a prioritized set of driver instructions. The environmental summary provides a concise overview of detected conditions. The risk factor analysis highlights specific hazards, such as excessive speed, inclement weather, or detected obstacles. Finally, the intervention delivers clear corrective actions.

The LLM dynamically adjusts the severity and urgency of alerts based on the detected risk level. Minor risks, such as light rain or moderate congestion, result in advisory messages. In contrast, high-risk situations such as speeding and low-visibility, trigger high-priority safety warnings. The resulting intervention is an auditory alert vocalized using text-to-speech systems.

Furthermore, the system updates interventions in real-time to reflect changing road conditions. If a previously detected risk factor is no longer present, the intervention is modified to prevent unnecessary driver stress. This adaptability distinguishes the framework from static rule-based warning systems, enhancing its effectiveness in promoting driver safety.

## 3 Results

Evaluating our framework involves assessing its efficiency in distracted driving scenarios, focusing on generating concise, accurate, real-time responses. We consider these performance indicators: alert correctness (AC), response latency, verbosity, false alarm, and missed detection rates.

### 3.1 Alert correctness

AC measures how accurately the system synthesizes multi-modal data into meaningful driver interventions. AC evaluates the retrieved data's factual accuracy and the system's ability to generate contextually relevant alerts. An alert is correct if it accurately reflects the driving environment's state based on agent outputs, ensuring that generated alerts are devoid of hallucinations and misinformation.

The correctness score is a weighted combination of semantic similarity and factual accuracy. Let **E**_*a*_*i*__ and **E**_*t*_*i*__ represent the embedding vectors of the system's generated intervention and the ground-truth environmental state with *F*_1_ denoting the factual accuracy score. The overall correctness score is defined as:


(1) $$\begin{array}{l} \text{Correctness}=\omega \cos\left({\text{E}}_{a_{i}},{\text{E}}_{t_{i}}\right)+\left(1-\omega \right)F_{1}, \end{array}$$

where the weighting parameter 0 ≤ ω ≤ 1 balances factual accuracy and contextual relevance. This evaluation compares system-generated interventions with ground-truth conditions across diverse driving scenarios. A high correctness score indicates effective integration of agent-derived data, while a lower score highlights low confidence in the intervention.

### 3.2 Latency of response generation

In real-time distracted driving intervention, measuring the time from distraction detection to alert issuance is crucial. The response latency metric quantifies how quickly the system processes environmental data and delivers driver alerts. Unlike conventional latency optimization, this evaluation examines how different modality configurations affect the overall response time rather than fine-tuning individual agent execution.

The total response latency, *T*_response_, is calculated as the sum of processing times across all stages:


(2) $$\begin{array}{l} T_{\text{response}}=T_{\text{LLM}}+T_{\text{agents}}+T_{\text{API-calls}}+T_{\text{delivery}}, \end{array}$$

where *T*_LLM_ is the time taken by the LLM to process the distraction signal and initiate agent calls, *T*_agents_ represents the execution time of the invoked agents, *T*_API-calls_ represents the time taken to receive a response from API calls, and *T*_delivery_ is the time to generate and transmit the intervention to the driver. Each agent's external API invocation and response acknowledgment expended ≈10 − 15ms.

Empirical studies indicate that a perception-reaction time of 2.5 seconds covers over 90% of drivers during unexpected braking scenarios (Federal Highway Administration Research and Technology, 2004). Therefore, setting *T*_max_ = 2 seconds ensures timely interventions, maintaining the system's effectiveness in mitigating driving risks. Exceeding this threshold may delay responses and reduce safety benefits.

### 3.3 Response efficiency and verbosity control

Effective intervention systems require clear and concise alerts, especially in safety-critical contexts. Controlling response verbosity ensures that interventions are easily understandable and actionable. Overly verbose responses can overwhelm drivers, delaying vital information and increasing cognitive load. To balance informativeness with brevity, the word count metric indicates response efficiency. Structured agent execution constrains verbosity, grounding responses within context and avoiding speculative reasoning.

The average word count per intervention, *W*_response_, across different agent configurations is calculated as:


(3) $$\begin{array}{l} W_{\text{response}}=W_{\text{LLM}}+W_{\text{agents}}, \end{array}$$

where *W*_LLM_ represents the words generated by the LLM after synthesizing agent outputs, and *W*_agents_ accounts for structured information from agents. Although no strict word limit for driver alerts exists, concise communication is crucial for safety. Setting *W*_max_ = 95 words serves as a practical guideline, ensuring interventions remain clear and concise. Exceeding this threshold hinders driver attentiveness, as excessive verbosity is impractical for real-time assistance. Standard text-to-speech systems take approximately 22 seconds to vocalize 95 words. To enhance alert effectiveness, the word “ALERT!” is prepended to each response, taking an additional ≈253 ms to vocalize, ensuring immediate driver attention.

### 3.4 False alarm and missed detection analysis

To comprehensively evaluate the system's reliability, we assessed the rates of false alarms (incorrect alerts) and missed detections (failure to recognize hazards) across all configurations. These metrics help determine the practical viability of real-time this system as excessive false alarms and missed detections can compromise driving safety.

The false alarm rate is the proportion of generated alerts that did not correspond to any verified driving hazard. The missed detection rate was determined as the proportion of unrecognized hazards among all ground-truth danger instances.

In our framework, *hallucinations* are defined as plausible-sounding but ungrounded LLM outputs that either fabricate environmental hazards not supported by any active agent (false alarms) or omit critical information that was correctly retrieved by an agent (missed detections). We trace each system-generated response back to its contributing agent outputs to assess this. If any part of the explanation cannot be mapped to a sensor stream or API response, it is flagged as a hallucination.

Our structured prompt template explicitly instructs the LLM to synthesize outputs strictly from agent data, not open-ended language priors. This grounding strategy curbs the generative model's tendency to extrapolate, ensuring that responses remain in a verified environmental context.

### 3.5 Evaluation setup

We utilized the lightweight version of the LLaMa3.2-1B LLM to evaluate the framework's performance, deployed on an A40-8Q GPU with 8GB of VRAM and 16GB of RAM. The LLM (LLaMa3.2-1B) required approximately 1.8GB of memory. On average, the multi-modal data processed per inference consisted of ≈110 tokens (80 words), including sensor inputs and agent outputs. We consider three baseline configurations to evaluate the impact of structured orchestration and agent integration. First, we use a pretrained LLaMA3.2-1B model (V-LLaMA) with unrestricted access to all agents but without structured prompts or task definitions (V-LLaMA w/o agent descriptions), serving as a baseline for ungrounded reasoning and undefined agent invocation. Second, we include a variant (V-LLaMA w/ agent descriptions) incorporating agent descriptions but omitting structured prompt templates, isolating the effect of instruction and task grounding. Third, we evaluate LLaMA3.2-11B-Vision (V-MM-LLaMA), a state-of-the-art multi-modal LLM capable of visual input processing, to assess whether a general-purpose vision-enabled model can match the performance of our domain-specific, agent-based framework in post-distraction intervention scenarios. These baselines allow us to analyze the independent and combined effects of agent access, descriptive grounding, and structured instruction prompting on response accuracy, task relevance, and overall intervention quality. Finally, we analyze the effect on including various combinations of agents throughout the framework when prompt grounding and agent descriptions are available.

A comprehensive evaluation of the proposed LLM-orchestrated intervention system was conducted using a diverse, multi-modal dataset to assess its correctness in generating driver interventions and efficiency in real-time operation. Our evaluation involved testing the system across seven distinct modality configurations to examine its ability to leverage awareness agents. We curated 100 diverse driving scenarios with varied conditions, producing 700 evaluated samples. Human-generated ground-truth interventions were meticulously crafted for each configuration to ensure rigorous testing. Additionally, we developed 35 validation Q&A pairs per configuration, totaling 245 instances, to probe the system's ability to generate appropriate interventions.

Regarding implementation feasibility, the proposed system is designed for onboard deployment within a vehicle rather than relying on remote processing. To ensure lightweight and efficient real-time operation, we employ both the lightweight versions of LLaMa and YOLO (total of < 3 GB of RAM). The multi-modal LLM is used as a multi-modal baseline, however, it is inefficient in deployment as this model is resource intensive, requiring at least 16GB of memory with a highly capable GPU. These capabilities may not always be available to vehicles.

### 3.6 Numerical results

The system was evaluated under seven combinations, varying the YOLO awareness (Y), the speed (S), traffic (T), and weather (W) awareness agents.

#### 3.6.1 Ablation study and correctness

To assess the contribution of each module in our framework, we conducted an ablation study across varying agent configurations, as summarized in Table 1. Results show that multi-agent setups consistently outperform single-agent configurations, confirming the value of diverse environmental inputs. Within individual agents, integrating fine-tuned YOLO (Y) to recognize traffic signs and speed limits, objects, and infer traffic conditions achieved the highest correctness scores of 81.2%. Integrating only the weather agent (W) induced the worst performance, as a distracted driving environment cannot be fully deduced from general weather information independently.

**Table 1:** Ablation study results for different agent combinations.

**Configuration**	**AC (%)**	**Latency (s)**	**Verbosity (# of words)**	**False alarm rate (%)**	**Missed detection rate (%)**
V-LLaMA w/o agent descriptions	68.3	>4.5	130+	12.1	12.5
V-LLaMA w/ agent descriptions	73.0	>3.0	100+	12.1	9.8
V-MM-LLaMA w/o agents	75.8	>8.3	120+	15.9	13.3
Y	81.2	1.42	58	8.7	6.4
S	67.2	1.12	33	9.3	7.1
T	62.7	1.20	50	10.5	8.3
W	60.4	1.27	47	11.0	8.9
Y + S	82.2	1.51	70	6.8	4.9
Y + T	82.0	1.54	66	7.1	5.4
Y + W	81.8	1.60	64	7.5	5.7
Y + S + T	84.4	1.65	74	5.3	3.7
Y + S + W	83.3	1.62	65	5.7	4.0
Y + T + W	82.8	1.62	63	6.0	4.3
Y + S + T + W	86.1	1.74	80	4.2	3.1

Among dual combination agents, the speed awareness module (Y+S) delivered the highest correctness at 82.2%, followed by traffic awareness (Y+T) at 82.0%, and weather awareness (Y+W) at 81.8%. When including three agents, including speed, traffic awareness with visual awareness (Y+S+T), achieved the highest correctness scores (84.4%) as the strongest environmental information is included to generate a reasoned and actionable alert. The full configuration (Y+S+T+W) achieved the highest correctness of 85.7%, demonstrating the effectiveness of fusing real-time perception, regulatory data, and external conditions to support robust intervention decisions.

We further compare our approach to baseline LLMs with varying degrees of agent access and prompting structure. V-LLaMA with agents but no agent descriptions achieved 68.3% correctness, highlighting the difficulty of effective reasoning without contextual cues. Providing agent descriptions raised performance to 73.0%, while introducing structured instruction templates and role definitions as core to our agentic framework further increased correctness by ensuring concise and task-aligned responses. The vision-enabled baseline V-MM-LLaMA achieved 75.8%. Despite leveraging multi-modal vision, this setup lacked real-time grounding and structured interaction, reducing clarity, increasing latency, and frequent off-topic reasoning. Additionally, the multi-modality of this model was tailored toward general visual summary tasks and not accurate object detection and classification tasks.

#### 3.6.2 Inference latency

Ensuring rapid response times is critical to maintaining driver attention. Our framework consistently demonstrates sub-2-second response times across all configurations. As additional modalities are introduced, the marginal increase in inference latency is an expected trade-off, as retrieving and processing multiple sensor modalities necessitates additional agent invocations. However, even the most computationally intensive case (Y+S+T+W) remains within the real-time threshold (1.74 s). With perception, the YOLO-only configuration is the fastest (1.42 s).

The V-LLaMA baselines exhibit prohibitively high inference latency, consistently exceeding 3.0 seconds and reaching over 4.5 seconds across all configurations, making them unsuitable for real-time distracted driving intervention. V-Multi-modal LLaMA, despite its vision capability, incurs even greater delays, with average response times exceeding 8.3 seconds. These delays stem from the absence of structured agent definitions, reliance on unconstrained reasoning over large token contexts, and the overhead of internal visual encoding. Unlike our agentic framework, which delegates targeted tasks to lightweight modules with minimal overhead, large multi-modal LLMs process the entire scene holistically, resulting in substantial computational burden and longer response generation times. Additionally, such models require powerful GPUs and extensive memory, limiting their feasibility for deployment in resource-constrained or edge-based driver assistance systems.

#### 3.6.3 Response efficiency and verbosity control

An optimal intervention must be informative yet concise, avoiding excessive verbiage that could overwhelm the driver while still conveying necessary corrective actions. The agent-integrated model maintains a controlled response length, averaging 64-80 words across all configurations, indicating that agent-based with structured prompt templates and reasoning effectively structures response synthesis without unnecessary elaboration.

By contrast, the baseline V-LLaMA and V-MM-LLaMa models produce significantly longer responses, with word counts exceeding 100. This verbosity is attributed to unconstrained generation without structured agent guidance, causing an over-explanation of conditions or speculative reasoning. Excessive verbosity increases cognitive load and contributes to longer inference times, compounding the delay in delivering timely interventions.

#### 3.6.4 False alarm and missed detection analysis

The analysis revealed that the multi-agent integrated setup (Y+S+T+W) achieved the lowest false alarm rate of 4.2% and a missed detection rate of 3.1%, yielding a combined hallucination-adjacent error rate of 7.3%. This analysis indicates a well-balanced performance in generating timely and relevant alerts. In contrast, single-agent configurations exhibited higher rates; for instance, the YOLO-only setup resulted in 8.7% false alarms and 6.4% missed detections. Unstructured baselines such as V-LLaMA without agent descriptions and V-MM-LLaMA produced significantly higher error rates, ranging from 24.6% to 29.2%.

Notably, the grounded configuration (Y+S+T+W) achieved the highest correctness (86.1%) and minimized hallucination-prone outputs. This result reinforces the effectiveness of our agent orchestration and prompt design, especially in contrast with V-MM-LLaMA and V-LLaMA variants, where the absence of grounding and structured instruction led to overly verbose, speculative, or misaligned outputs. These results substantiate the role of agent grounding in enhancing factual accuracy, minimizing misfires, and maintaining safety-critical reliability in post-distraction driver interventions.

### 3.7 Case studies

To showcase the framework's effectiveness under diverse real-world conditions, we demonstrate its performance across three distinct driving environments: clear, busy, and obscure conditions. Each scenario presents unique challenges that test the system's ability to synthesize multi-modal environmental data and generate contextually appropriate interventions.

#### 3.7.1 Clear conditions

**Figure 2:**
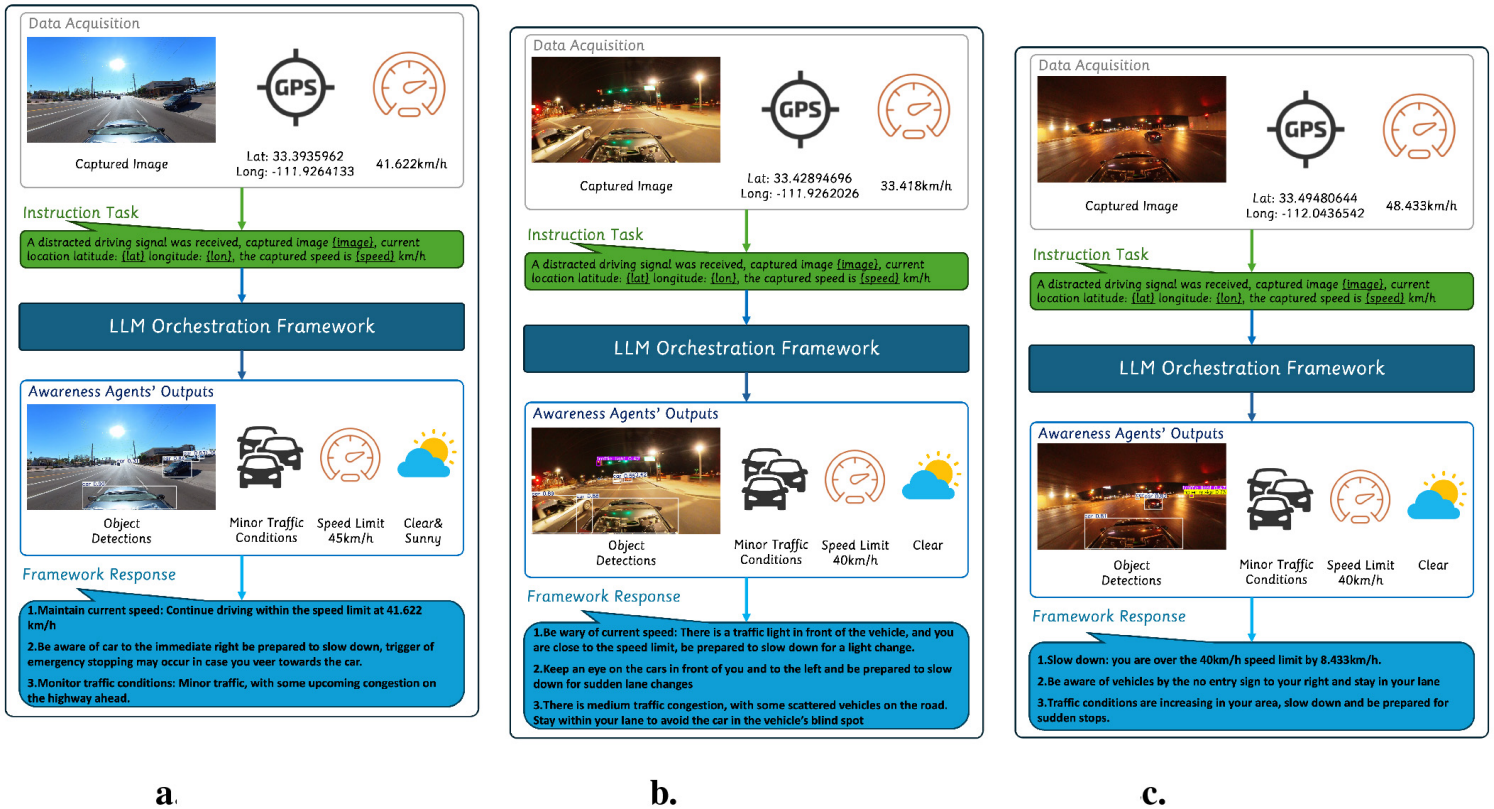
Case study showing system responses in different driving conditions. **(a)** Speed compliance and minor congestion. **(b)** Signal and traffic monitoring. **(c)** Violation detection in low visibility.

In clear driving conditions, where visibility is unobstructed with minimal environmental disruptions, the system primarily focuses on maintaining compliance with traffic regulations and reinforcing situational awareness. In the scenario shown in Figure 2a, the system successfully detected a vehicle positioned perpendicularly to the roadway, exiting a parking lot. The response effectively balanced speed compliance, proximity awareness, and real-time traffic monitoring.

The system verified that the vehicle was traveling within the legal speed limit and reassured the driver to maintain their current speed. On recognizing a vehicle to the right, the system issued a cautionary advisory, instructing the driver to remain alert and slow down if necessary. Additionally, the system preemptively warned on intervening with the emergency braking mechanism in case of an abrupt lane change toward the detected vehicle. The structured response ensures that only relevant insights are communicated, reinforcing safe driving behavior while minimizing cognitive overload.

#### 3.7.2 Busy conditions

High-traffic environments introduce dynamic risks that require the system to balance situational awareness, regulatory compliance, and proactive intervention. The scenario shown in Figure 2b illustrates the system's ability to generate adaptive responses suited for complex road conditions.

The system first determined that the driver was traveling below the posted speed limit. On detecting an upcoming traffic light, the system advised the driver to stay vigilant and be prepared to slow down for a light change.

Beyond speed awareness, the system detected multiple vehicles in the surrounding environment, advising the driver to remain aware of nearby cars. The system recognized the potential for sudden lane changes and preemptively warned the driver to anticipate movement from nearby vehicles. Additionally, the system identified and incorporated real-time congestion analysis, identifying moderate traffic density with scattered vehicles. The response provided a specific recommendation, instructing the driver to remain within their lane to avoid a vehicle in their blind spot.

#### 3.7.3 Obscure conditions

Nighttime driving and low-visibility environments pose challenges for visual perception systems due to limited lighting and occluded road elements. As shown in Figure 2c, the driver navigates an underpass at night. In low-visibility scenarios, structured regulatory enforcement becomes crucial. Despite these conditions, the system successfully processed the data to generate a meaningful intervention. The system accurately identified a speed limit violation that exceeded the limit by 8.4 km/h and issued a speed reduction advisory.

Additionally, the system provided a proximity alert regarding vehicles near a no-entry sign detected to the right. Although the detection process resulted in a conflict between a no-entry sign and a traffic light, the system maintained high contextual accuracy by prioritizing nearby vehicle positioning over ambiguous signage interpretations.

The system also detected an increase in traffic density within the area. Recognizing the potential for sudden stops, the intervention advised the driver to reduce speed and remain prepared for abrupt braking events.

## 4 Discussion

### 4.1 Challenges

While the proposed LLM-agentic orchestration framework demonstrates strong performance across multiple metrics, several technical challenges must be addressed for robust deployment in real-world vehicular systems.

#### 4.1.1 Edge deployment and resource constraints

Despite using compressed models (YOLOv11 and LLaMA3.2-1B), real-time orchestration of multi-modal agents imposes non-trivial computational loads. Running inference pipelines concurrently for object detection, LLM reasoning, and external API queries requires efficient scheduling on low-power vehicle-grade edge processors (e.g., NVIDIA Jetson, Qualcomm Snapdragon Ride). Balancing response latency and power consumption remains a critical bottleneck, particularly under high sensor input frequency or multi-agent invocation chains.

#### 4.1.2 Dependency on external APIs and network latency

The proposed framework leverages external APIs to supplement perception gaps and enhance intervention accuracy. While these agents significantly enrich contextual awareness, their effectiveness depends on stable and low-latency internet connectivity. In real-world driving scenarios, particularly in rural, underground, or high-interference zones, API access may be delayed or disrupted entirely due to network instability, limited bandwidth, or data plan restrictions.

To mitigate such issues, the framework prioritizes local perception through the YOLO agent, which serves as the primary and default sensing mechanism. APIs are invoked only when the LLM identifies ambiguity or missing environmental context. For example, the system only calls corresponding agents if speed limit signs are not visually detected or traffic density patterns are unclear. This supplementary action ensures graceful degradation: even without external data, the system continues to function autonomously based on local sensor cues.

Nevertheless, reliance on API calls introduces challenges such as variable latency, rate limiting, or complete outages. These limitations necessitate the design of robust fallback strategies, including: (1) caching previously queried results, (2) preloading map and weather data in known high-risk areas, (3) integrating lightweight onboard models as surrogates for API agents, and (4) adopting hybrid edge-cloud inference pipelines. These enhancements ensure the system maintains situational awareness, minimizes response latency, and sustains reliable performance in diverse deployment environments.

#### 4.1.3 Agent invocation logic and redundancy

The agent evaluator follows a rule-based invocation logic, which, while modular, lacks adaptability. This shortcoming can result in suboptimal agent combinations or unnecessary agent calls in low-risk scenarios. Moreover, redundancy across agents (e.g., YOLO detecting speed signs while the speed awareness gent queries GPS-based limits) may cause information overlap and inefficient resource utilization. A more intelligent agent selection policy that dynamically weighs the utility of agent outputs is needed.

#### 4.1.4 Hallucination risk and contextual overfitting

Although structured prompts, prompt engineering, and grounded agent responses reduce hallucinations, LLMs can still generate speculative or contextually misaligned outputs when encountering ambiguous or conflicting agent data. Fine-grained control over reasoning steps through intermediate verification modules or constrained decoding techniques remains an open challenge for ensuring reliability. Mitigations for this technique include keeping track of previous responses to act as a history for expected response behavior through long-term memory.

### 4.2 Conclusion

We introduce a multi-modal agentic LLM framework to enhance driver safety through real-time, context-aware interventions. Our approach leverages agentic reasoning to process diverse data and generate actionable alerts for distracted driving scenarios. Evaluations show that integrating multiple agents with structured prompts improves accuracy, and enhances efficiency. Full agent integration achieved the highest correctness (85.3%), with speed and traffic awareness being critical in standard conditions and weather awareness essential in adverse scenarios. The system consistently meets the sub-2s latency requirement, outperforming V-LLaMA in both latency and accuracy. These findings underscore the potential of LLM-driven multi-modal reasoning for AI-assisted driver support. Future work focuses on incorporating additional sensory data, adaptive driver monitoring, and improved agent selection through reinforcement learning.

### 4.3 Future work

Building upon the proposed LLM-driven agentic intervention framework, several research directions can further enhance its robustness, scalability, and real-world applicability.

#### 4.3.1 Reinforcement learning for agent scheduling

Future iterations will explore using reinforcement learning (RL) to optimize agent invocation strategies. Rather than relying on fixed rules, an RL-based policy could learn to select the minimal yet sufficient agents required to meet correctness and latency constraints. This approach can dynamically balance performance and resource usage under varying environmental and computational conditions.

#### 4.3.2 On-Device knowledge graphs for offline reasoning

We plan to construct on-device, compressed knowledge graphs encompassing traffic rules, weather patterns, and map data to reduce reliance on external APIs. These will be queried using LLM-based reasoning locally through retrieval-augmented generation (RAG), minimizing latency and improving robustness under weak connectivity. Integration with tools like Facebook AI Similarity Search (FAISS) and quantized vector stores will be explored for efficient memory usage.

#### 4.3.3 Integration of low-level vehicle telemetry

The current framework focuses on external perception and driver distraction. Future work will incorporate CAN bus data, including acceleration, braking, and steering signals, to infer driver intent and vehicle state. Fusing high-frequency telemetry with semantic LLM-driven reasoning opens new opportunities for proactive intervention and richer situational grounding.

#### 4.3.4 Multi-agent coordination and temporal planning

Extending beyond single-turn decision-making, we aim to implement temporally extended reasoning via hierarchical agent planning. This step includes inter-agent memory, trajectory forecasting, and attention modulation based on recent environmental dynamics. Agents could operate asynchronously, leveraging buffer queues and task prioritization to maintain responsiveness.

## Data Availability

The original contributions presented in the study are included in the article/supplementary material, further inquiries can be directed to the corresponding author.

## Footnotes

^1^HERE API: https://www.here.com/platform/map-data.

^2^OpenMeteo API: https://open-meteo.com/en/docs.

^3^OpenStreetMap API: https://wiki.openstreetmap.org/wiki/Overpass_API.
